# The essential and downstream common proteins of amyotrophic lateral sclerosis: A protein-protein interaction network analysis

**DOI:** 10.1371/journal.pone.0172246

**Published:** 2017-03-10

**Authors:** Yimin Mao, Su-Wei Kuo, Le Chen, C. J. Heckman, M. C. Jiang

**Affiliations:** 1 Applied Science Institute, Jiangxi University of Science and Technology, Jiangxi, China; 2 Department of Physiology, Northwestern University, Chicago, Illinois, United States of America; 3 Department of Physical Medicine and Rehabilitation, Northwestern University, Chicago, Illinois, United States of America; 4 Department of Physical Therapy and Human Movement Sciences, Northwestern University, Chicago, Illinois, United States of America; Institute of Health Science, CHINA

## Abstract

Amyotrophic Lateral Sclerosis (ALS) is a devastative neurodegenerative disease characterized by selective loss of motoneurons. While several breakthroughs have been made in identifying ALS genetic defects, the detailed molecular mechanisms are still unclear. These genetic defects involve in numerous biological processes, which converge to a common destiny: motoneuron degeneration. In addition, the common comorbid Frontotemporal Dementia (FTD) further complicates the investigation of ALS etiology. In this study, we aimed to explore the protein-protein interaction network built on known ALS-causative genes to identify essential proteins and common downstream proteins between classical ALS and ALS+FTD (classical ALS + ALS/FTD) groups. The results suggest that classical ALS and ALS+FTD share similar essential protein set (VCP, FUS, TDP-43 and hnRNPA1) but have distinctive functional enrichment profiles. Thus, disruptions to these essential proteins might cause motoneuron susceptible to cellular stresses and eventually vulnerable to proteinopathies. Moreover, we identified a common downstream protein, ubiquitin-C, extensively interconnected with ALS-causative proteins (22 out of 24) which was not linked to ALS previously. Our *in silico* approach provides the computational background for identifying ALS therapeutic targets, and points out the potential downstream common ground of ALS-causative mutations.

## Introduction

Amyotrophic Lateral Sclerosis (ALS) is a neurodegenerative disorder characterized by progressive and selective loss of upper and lower motoneurons with no effective treatment available[1]. Clinical symptoms includes tremor, muscle weak, spasticity and paralysis, and patients usually die from respiratory failure within five years[2]. The majority (90–95%) of ALS patients are sporadic form (sALS), only small cohort of patients (5–10%) are associated to autosomal dominant inheritance as familial cases (fALS)[3, 4]. The incidence rate is at 2.7/100,000 in a ten-year Ireland research[5]. To date there are 26 subtypes of ALS listed in the Online Mendelian Inheritance in Man (OMIM) database with varied disease onset time and symptom onset origin, and 15–52% ALS patients are comorbid with Frontotemporal Dementia (FTD)[6, 7], the second most common dementia representing series of neurological symptoms involving frontotemporal lobar degeneration. FTD can exist alone without developing ALS, while certain forms of FTD and ALS shared some clinical and genetic features [8].

Although the pathogenic mechanisms of ALS are not fully clear, a number of gene mutations linked to ALS were discovered over past 20 years, such as superoxide dismutase 1 (*SOD1*), TAR DNA-binding protein (*TARDBP*), fused in sarcoma (*FUS*), optinurin (*OPTN*), valosin-containing protein (*VCP*), sequestosome-1 (*SQSTM1*), ubiquilin-2 (*UBQLN2*), *C9ORF72*, heterogeneous nuclear ribonucleoproteins A1 and A2/B1 (*HNRNPA1* and *HNRNPA2B1*)[9–18]. In particular, SOD1, TDP-43, FUS, optinurin and ubiquilin-2 proteins were identified in aggregates from the autopsies of many patients[19]. These ALS-causative genes encoded proteins with divergent functions, many of which can be related to several categories, such as cellular transport (axonal/vesicle transport or whose aggregation would impede transport), RNA processing and ubiquitin proteasome system. However, it remains unclear how these minimally related proteins, once impaired, all result in motoneuron degeneration which eventually lead to various ALS subtypes. One explanation is that some of the ALS-causative proteins are “essential proteins” that, when acted inappropriately on by other ALS-causative mutations, would result in profound effects to motoneuron survival. An alternative hypothesis is that there may be common downstream proteins, which maximally connect with those ALS-causative proteins through either direct or indirect interaction.

The original idea of “essential proteins” is that these proteins are crucial for survival and that their deletion confers the lethal phenotype[20]. The conventional experimental approaches in identifying essential proteins, such as gene knock-out or RNA interference, are usually time-consuming and cost-intensive. Several previous studies have shown the feasibility of computational approaches to predict gene essentiality and morbidity[21–24]. For example, topological properties of protein-protein interaction (PPI) have been employed to identify essential proteins in various organisms[25, 26]. The main idea is the “centrality-lethality rule”, in which highly connected hub proteins are more essential to survival in PPI network[22]. Although there is still significant debate regarding the rule, several studies suggest a correlation between topological centrality and protein essentiality[27–29]. To identify crucial ALS-causative proteins, we applied this same concept to calculate the essentiality of each protein in a PPI network. This approach also allowed us to investigate the roles of ALS/FTD mutations such as C9ORF72 in the PPI network, which had been shown to involve distinct pathways from sALS in brain transcriptome[30].

Given such divergent functional background of these ALS mutations, it is tempting to hypothesize that there might be a common downstream interaction, either via individual proteins or convergent pathways, which would render motoneuron vulnerable to toxicity. To evaluate this possibility, we fully explored the ALS PPI network to analyze those proteins capable of interacting with ALS-causative proteins directly. If such proteins do exist, they must maximally interact with ALS-causative proteins and play vital roles in maintaining cellular activities. Thus upstream ALS-causative mutations might impair or disrupt the function of downstream proteins and cause long-term toxic effects.

In current study, we constructed a PPI network based on ALS-causative genes imported from the OMIM database. Genes linked only to classical ALS were grouped with or without genes implicated in ALS/FTD, namely ALS+FTD group vs. classical ALS group. Our integration of network topological properties and protein cluster information revealed that classical ALS and ALS+FTD groups showed similar essential protein sets (VCP, FUS, TDP-43 and hnRNPA1) but distinct patterns in functional enrichment analysis. These essential proteins might present a set of proteins susceptible to disruptions. Moreover, we identified an interconnected common protein, ubiquitin-C, which extensively interacts with almost all ALS-causative proteins (22 out of 24 proteins). To the best of our knowledge, ubiquitin-C has not been linked to ALS yet. Our results, based on computational analyses, suggest potentials of novel disease mechanisms that may underlie various forms of ALS and shed light on new direction in ALS study

## Materials and methods

**Fig 1:**
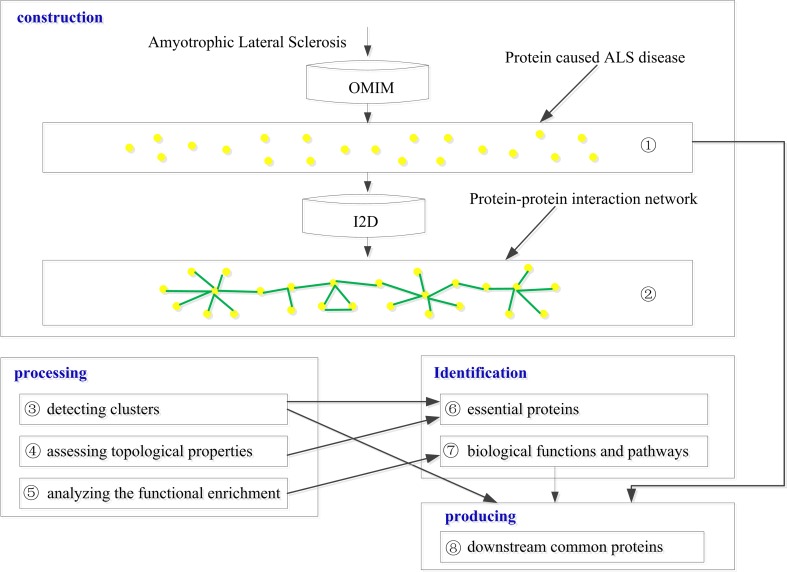
Overall work flow.

The overall workflows used in this study are shown in Fig 1. The process involves four main steps: construction, processing, identification and producing. Construction consisted of obtaining ALS causal genes from OMIM database (labeled (1) in Fig 1) and constructing the protein-protein interaction network of ALS based on I2D database (2). Processing consisted of detecting clusters by analyzing PPI (3); assessing topological properties by measuring degree centrality in the network based on global PPI (4); and finally, analyzing functional enrichment and pathway (5). Identification consisted of: finding essential proteins for ALS by adopted edge clustering coefficient (ECC) method (6) and identifying the biological functions and pathways by GO and KEGG pathway enrichment analysis (7). Producing consisted of analyzing downstream common proteins by designed DFloyd algorithm (8).

### 2.1 Genetic defects in ALS and construction of PPI network

OMIM database is a disease phenotype database and a catalogue of human genes and genetic disorders[31]. In OMIM, the list of hereditary disease genes is described in the OMIM morbid map. The ALS-causative genes were imported from the OMIM morbid map (https://www.omim.org/phenotypicSeries/PS105400). Corresponding protein names and IDs were obtained by investigating the mapping scheme of UniProt database[32]. Hence we only considered those loci with known encoding protein profiles in UniProt, thus ALS3 and ALS7 were excluded in this research. We referred to genes linked to ALS1-22 in OMIM, except ALS3 and ALS7, as classical ALS (20 genes); FTDALS 1–4 were regarded as ALS/FTD (4 genes) (Table 1). Total ALS-causative genes were referred to ALS+FTD (20+4 genes) in this research. In order to investigate the differences between classical ALS and ALS+FTD groups, we explored experimentally validated interactions from the Interologous Interaction Database (I2D) database (http://ophid.utoronto.ca/ophidv2.204/)[33, 34]. I2D is a protein-protein interaction database and an integrated database of known experimental and predicted human protein interaction data sets (including HRPD, BIND, and BioGrid). The gene names were inputted into I2D database with human as the only chosen target organism. I2D contains more than 290,000 experimental interactions from 38 databases of human source[34]. Thus, choosing I2D instead of single database might help minimizing the bias[35]. All homologous predicted protein interactions in I2D database were excluded to increase the reliability of protein interaction data. The rest experiment-based PPIs were then used to construct classical ALS and ALS+FTD PPI networks with ALS-causative proteins/interacting partners as nodes and interactions as edges. The comprehensive interaction data published in the I2D database enabled our analysis on a complete network of disease proteins. Identifiers of proteins were unified using the protein IDs defined in the UniProt database[32]. Some proteins were given multiple names, to avoid the ambiguous referring, the results in tables and figures were presented in the format of gene name and UniProt ID. Our study considered the I2D database version 2.9 released in September 2015.

**Table 1:** ALS-causative genes from OMIM.

Subtype	Uniprot ID	Gene	Subtype	Uniprot ID	Gene	Subtype	Uniprot ID	Gene
ALS1	P00441	*SOD1*	ALS10	Q13148	*TARDBP*	ALS19	Q15303	*ERBB4*
ALS2	Q96Q42	*ALS2*	ALS11	Q92562	*FIG4*	ALS20	P09651	*HNRNPA1*
ALS3	-	*-*	ALS12	Q96CV9	*OPTN*	ALS21	P43243	*MATR3*
ALS4	Q7Z333	*SETX*	ALS13	Q99700	*ATXN2*	ALS22	P68366	*TUBA4A*
ALS5	Q96J17	*SPG11*	ALS14	P55072	*VCP*	-----------------------------------------		
ALS6	P35637	*FUS*	ALS15	Q9UHD9	*UBQLN2*	FTD-ALS1	Q96LT7	*C9ORF72*
ALS7	-	*-*	ALS16	Q99720	*SIGMAR1*	FTD-ALS2	Q8WYQ3	*CHCHD10*
ALS8	O95292	*VAPB*	ALS17	Q9UQN3	*CHMP2B*	FTD-ALS3	Q13501	*SQSTM1*
ALS9	P03950	*ANG*	ALS18	P07737	*PFN1*	FTD-ALS4	Q9UHD2	*TBK1*

### 2.2 Computing topological properties of protein interaction network

We evaluated the centrality of proteins in PPI network to investigate the essentiality of proteins. Many research works indicated that PPI networks have characters of “small-world behavior” and “centrality-lethality” [22, 36]. Removal of nodes with high centrality makes the PPI network collapse into isolated clusters which might imply the collapse of biological system. Degree centrality of a protein indicates how many interactions that protein has to other proteins. For each node (protein), we applied the topological measures to assess its role in the network: degree centrality (*DC*). A PPI network is represented as an undirected graph *G* (*V*, *E*) with proteins as nodes and interactions as edges. The degree centrality is calculated by:

DC(v)=|e(u,v)|u,v∈V (Eq 1)

*v* represents a node in PPI network where *u* is any node other than *v* in the network. *e*(*u*, *v*) represents the interaction between *v* and *u*. If such an interaction does exist, the value of *e*(*u*, *v*) is one. If not, is *e*(*u*, *v*) zero. |*e*(*u*, *v*)| represents total interaction numbers between *v* and *u*.

### 2.3 Identification of clusters

A unique feature of the clique percolation clustering method (CPM) is that it can uncover the overlapping community structure of complex networks, i.e., one node can belong to several communities[37]. In order to detect the densely connected regions in network with possible overlap and their functions in the network, the PPI information data were imported into CFinder-2.0.6 (an open source software platform in CPM method), and the clustering analysis was easily performed. The clusters were identified to have the minimum k-cliques, which was defined as the union of all k-cliques (complete sub-graph of size k) that could be reached from each other.

### 2.4 Finding essential proteins

The use of global centrality measures based on network topology has become an important method in identifying essential proteins. However recent research pointed out that many essential proteins have low connectivity and are difficult to be identified by centrality measuring[37–41]. Hart *et al*. pointed out that clusters have high correlation with essential proteins[42]. A new method ECC was proposed to identify essential proteins by integration of PPI network topology and cluster information. The unabridged ECC method can be found in Ren *et al*.[43]. Its basic concepts are as follow.

In-degree *K*^*in*^ (*i*,*c*) of a protein *i* in a cluster *C* was defined as the number of interactions which connect *i* to other proteins in *C*.

$$K^{in}(i,c)=|\{e(i,j)|i,j\in v(v)\}|$$ (Eq 2)

The complex centrality of a protein *i*, *Complex_C(i)*, was defined as the sum of *in-degree* value of *i* in all clusters which included it. *Complex_C(i)* could define the overlapping number of protein complexes.

$$Complex\_C(i)=\sum_{C_{i}\in CS\&i\in C_{i}}K^{in}(i,C_{i})$$ (Eq 3)

Where *CS* was the cluster set, and *Ci* was a cluster which included *i*.

The global centrality adopted subgraph centrality (*DC*) as it had better performance in identifying essential proteins in centrality methods[29]. To integrate *DC*(*i*) and *Complex_C*(*i*), a harmonic centrality (*HC*) of protein *i* was defined as follows:

$$HC(i)=\alpha \times DC(i)/{DC}_{max}+(1-\alpha )\times Complex\_C(i)/Complex\_C_{max}$$ (Eq 4)

Where *α* was a proportionality coefficient and took value in range of 0 to 1, generally set 0.5, *DC*_*max*_ was the maximum *DC* value. *Complex_C*_*max*_ was the maximum *Complex_C* value.

### 2.5 Functional enrichment analysis

Functional enrichment analysis was performed to further study the functions and enriched pathways of cluster based on GO database (Version No.2010.09.03) (http://www.genontology.org/)[44] and KEGG pathway (http://www.genome.jp/kegg/)[45], respectively. In functional analysis, *P*<0.01 were considered statistically significant. This analysis was performed by using the database for annotation, visualization, and integrated discovery (DAVID, http://david.abcc.ncifcrf.gov/tools.jsp)[46, 47], which is an online platform providing functional annotation tools to analyze biological meaning behind large list of genes.

### 2.6 Downstream common protein analysis

We carried out five steps to analyze the downstream common proteins by designing a deformation Floyd (DFloyd) algorithm[34] of the shortest path. Give the source sets *S* and target *T*, *S* is the protein set that composed of two sources: (1) k-clique cluster proteins under highest possible k value excluding ALS-causative proteins; (2) proteins presented in significant enrichment analysis GO term and KEGG pathway. *T* is the ALS-causative protein set. The proteins in *S* and *T* are all part of ALS PPI Network. The algorithm of DFloyd is shown below.

Step1 Calculate the relation edge set W from source set S to target set T by the enumerating method. *W* = {*<s*_*1*_, *t*_*1*_*>*,*<s*_*1*_, *t*_*2*_*>*,*…*,*<s*_*1*_, *t*_*n*_*>*,*…*,*<s*_*n*_, *t*_*1*_*>*,*…*,*<s*_*n*_, *t*_*n*_*>*}
Step 2 Computer all distance(*s*_*i*_, *t*_*i*_), < *s*_*i*_, *t*_*i*_ > ∈*W*. If there is not an edge between *s*_*i*_ and *t*_*i*_, then distance(*s*_*i*_, *t*_*i*_) = ∞, else distance(*s*_*i*_, *t*_*i*_) = 1.
Step 3 Remove all *s*_*i*_, *t*_*i*_ from *W*, *s*_*i*_, *t*_*i*_ ∈distance(*s*_*i*_, *t*_*i*_) = 1
Step 4 For any < *s*_*i*_, *t*_*i*_ > in *W*, Label = False.
  If there is a vertax *v*, distance(*s*_*i*_, *ν*)+distance(*ν*, *t*_*i*_)< distance(*s*_*i*_, *t*_*i*_),
  then distance(*s*_*i*_, *t*_*i*_) = distance(*s*_*i*_, *ν*)+distance(*ν*, *t*_*i*_).
  Label = Ture
  Endfor
Step 5 Repeat step 4, until Label = False

### 2.7. Statistic

Thompson Tau test was performed to detect if the value was significantly deviated from mean value. Modified Thompson Tau was calculated as below:

$$\tau =\frac{t∙(n-1)}{\sqrt{n}∙\sqrt{n-2+t^{2}}}$$

t = Student’s t value, α = 0.05, df = n-2; ** >mean ±τ SD; * >mean ±(τ SD)/2

## Results

### 3.1 Genes and PPI

Genetic deficits accounting for various classical ALS (ALS1-2, 4–6 and 8–22; 20 genes) and ALS/FTD phenotypes (ALS/FTD1-4; 4 genes) were obtained from the OMIM database as shown in Table 1. We studied their interactions based on known protein-protein interactions by exploring the I2D database. To investigate the roles of ALS/FTD mutations in ALS, mutations linked to classical ALS phenotypes were separated from those causing ALS/FTD. The PPI network was constructed from proteins encoded by classical ALS (20) or ALS+FTD gene set (20+4). The I2D contains more than 230,000 experiment-based interactions and around 70,000 predicted interactions from human source. Our input of ALS-causative proteins into I2D yielded 4,144 interactions in classical ALS group and 5,454 interactions in ALS+FTD group. After removal of homologous predicted interactions, 3,023 (S1 Text) and 3,764 (S2 Text) interactions with 1,932 and 2,288 interacting nodes were used to construct the PPI networks of classical ALS and ALS+FTD respectively.

### 3.2 Network centrality degree analysis

The topological properties of protein interactions were calculated to identify essential proteins in the network. The degree centrality of each protein in PPI was ranked in Table 2. The majority of proteins with high degree centrality were proteins encoded by ALS-causative genes listed in Table 1, which was not surprising. Interestingly ubiquitin-C (UBC) and YWHAE presented in ALS+FTD group, as the only two proteins not encoded by ALS-causative genes. Both UBC and YWHAE have not been linked to ALS or FTD yet. Among both groups, VCP, hnRNPA1 and FUS were all in the top five with obviously higher degree centrality. The results suggested the importance and extensive involvement of VCP, hnRNPA1 and FUS in ALS pathogenesis. Moreover our results revealed an intriguing role of UBC in ALS+FTD PPI networks, a link that might have been otherwise overlooked. It is not surprising to see similar results between two groups because degree centrality lack of the topological information. Thus we performed cluster analysis to further examine the PPI network.

**Table 2:** Degree centrality ranking in ALS PPI network.

Classical ALS				ALS+FTD							
Rank	Uniprot	Protein	DC	Rank	Uniprot	Protein	DC	Rank	Uniprot	Protein	DC
1	P55072	VCP	729**	1	P55072	VCP	729**	15	Q15303	ERBB4	68
2	P35637	FUS	385*	2	Q13501	SQSTM1	596**	16	Q99700	ATXN2	55
3	P09651	HNRNPA1	351	3	P35637	FUS	385*	17	Q9UQN3	CHMP2B	27
4	Q13148	TARDBP	319	4	P09651	HNRNPA1	351	18	Q7Z333	SETX	25
5	P68366	TUBA4A	223	5	Q13148	TARDBP	319	19	P0CG48	UBC	22
6	P00441	SOD1	216	6	P68366	TUBA4A	223	20	Q96Q42	ALS2	14
7	P43243	MATR3	149	7	P00441	SOD1	216	21	Q96LT7	C9orf72	13
8	P07737	PFN1	110	8	P43243	MATR3	149	22	P62258	YWHAE	11
9	Q96CV9	OPTN	101	9	Q9UHD2	TBK1	144				
10	Q99720	SIGMAR1	90	10	P07737	PFN1	110				
11	Q9UHD9	UBQLN2	78	11	Q96CV9	OPTN	101				
12	O95292	VAPB	73	12	Q99720	SIGMAR1	90				
13	Q15303	ERBB4	68	13	Q9UHD9	UBQLN2	78				
14	Q99700	ATXN2	55	14	O95292	VAPB	73				

** >mean ±τ SD;

* >mean ±(τ SD)/2

### 3.3 Cluster analysis

**Fig 2:**
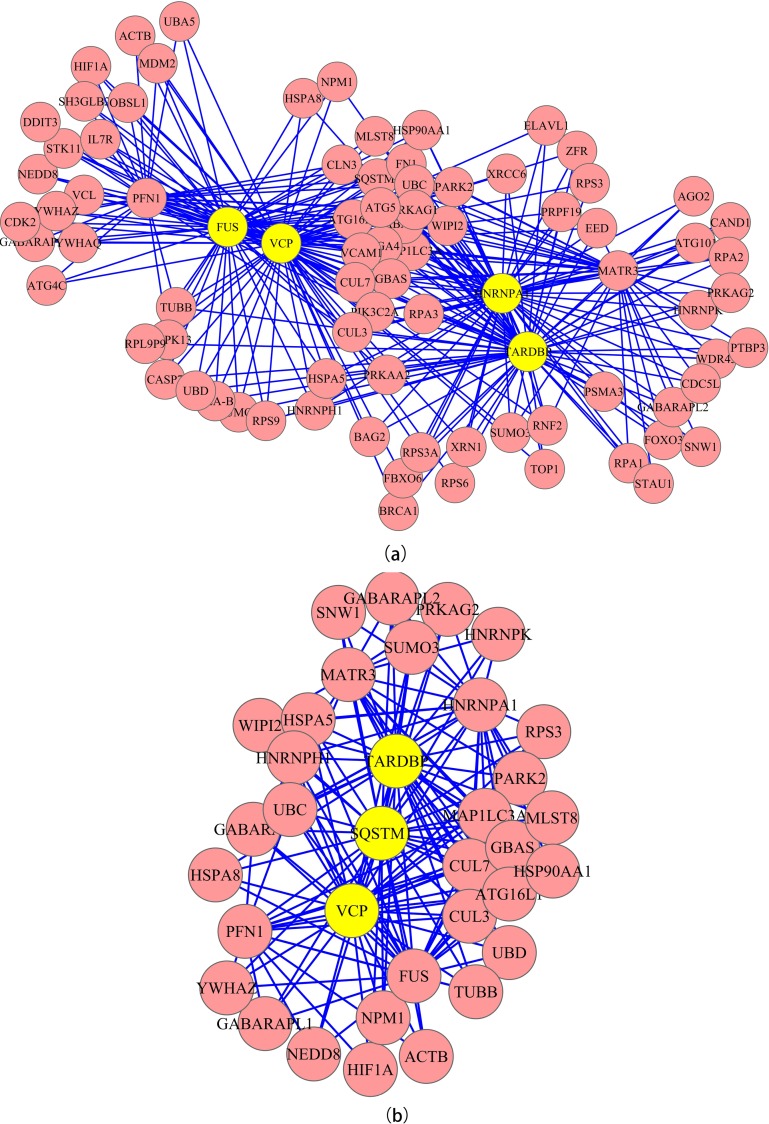
The clusters of (a) classical ALS, 136 clusters at k = 4; (b) ALS+FTD, 59 clusters at k = 5. The yellow highlights the core clusters identified by significant involvement ranking calculated in Table 3 based on Thompson Tau test.

To further scrutinize the complex ALS PPI network, we conducted a cluster analysis to get a clear picture of interactions between the proteins in the PPI. The clusters (cliques) with densely connected nodes in the PPI network were detected using the ClusterOne plug-in of the CFinder 2.0.6 software. In the classical ALS group, 393 and 136 clusters were identified with parameters set to a minimum size of three (k = 3) and four (k = 4) respectively, while in the ALS+FTD group, 578 clusters were detected when k = 3 and 59 clusters were found when k = 5. In current study VCP, FUS, hnRNPA1 and TDP-43 were the cores of network in classical ALS (k = 4); ALS+FTD (k = 5) groups shared similar feature but included SQSTM-1 instead of hnRNPA1 and FUS (Fig 2A and 2B). In the classical ALS group, however, a few proteins not encoded by classical ALS gene set appeared in the list: UBC, YWHAE, YWHAZ and sequestosome-1/p62 (SQSTM1). Mutations on *SQSTM1* are known to associate with ALS/FTD; In this research, *SQSTM1* was categorized in ALS+FTD group, but it also involved in some ALS without FTD cases[48], which directly validated our algorithm and highlighted the importance of SQSTM1/p62 in pathology across ALS-FTD spectrum. More importantly those surrounding proteins which interacted with the cluster cores provided important clues to further investigate downstream pathways of ALS pathogenesis. The involvement of each protein was assessed by calculating the number of clusters each protein participated in. VCP, TDP-43 and hnRNPA1 were at the top of the list in both groups (Table 3). Notably, UBC and YWHAE presented in both groups again as proteins not encoded by ALS-causative genes.

**Table 3:** Protein involvement based on participated cluster number.

Classical ALS				ALS+FTD			
Rank	Uniprot	Protein	Clusters	Rank	Uniprot	Protein	Clusters
1	P55072	VCP	296**	1	Q13501	SQSTM1	460**
2	Q13148	TDP-43	269**	2	P55072	VCP	351*
3	P09651	HNRNPA1	214*	3	Q13148	TDP-43	294*
4	P35637	FUS	185*	4	P09651	HNRNPA1	241
5	P43243	MATR3	81	5	P35637	FUS	228
6	P07737	PFN1	65	6	P43243	MATR3	88
7	Q96CV9	OPTN	25	7	P07737	PFN1	65
8	O95292	VAPB	20	8	P68366	TUBA4A	62
9	Q99700	ATXN2	16	9	P00441	SOD1	47
10	P0CG48	UBC	11	10	Q96CV9	OPTN	47
11	Q96Q42	ALS2	9	11	O95292	VAPB	20
12	P00441	SOD1	8	12	Q99700	ATXN2	16
13	Q13501	SQSTM1	8	13	P0CG48	UBC	14
14	Q9UHD9	UBQLN2	6	14	Q9UHD2	TBK1	12
15	P62258	YWHAE	6	15	Q96Q42	ALS2	8
16	P63104	YWHAZ	6	16	P62258	YWHAE	8

** >mean ±τ SD;

* >mean ±(τ SD)/2

### 3.4 Essential proteins

**Fig 3:**
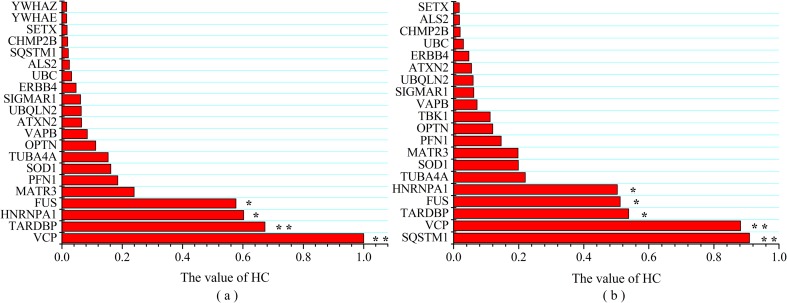
Essential proteins ranked by HC (a) classical ALS; (b) ALS+FTD. ** >mean ±τ SD; * >mean ±(τ SD)/2

**Fig 4:**
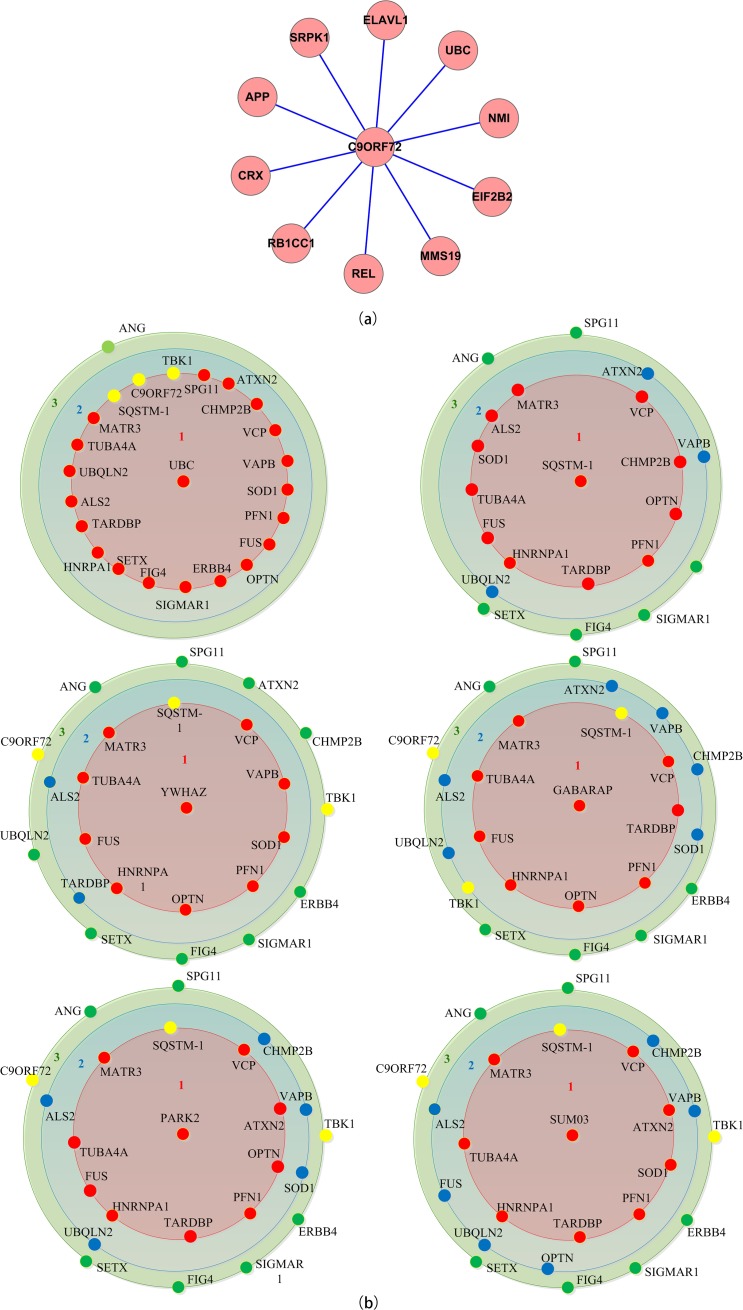
Profiles of the interacting proteins. A) Profile of C9orf72 with only direct interaction B) The profiles include both direct and indirect interactions of important downstream proteins. Red, blue and green denote direct, secondary and tertiary contact proteins respectively. Yellow highlights ALS/FTD proteins.

All above analysis were based on network topological properties. In order to take the relevance between interactions and protein essentiality into account, we used the ECC method to identify essential proteins in network. The result of harmonic centrality are shown in Fig 3 in which VCP, hnRNPA1, FUS and TDP-43 were the top-ranking hub proteins in both the classical ALS and ALS+FTD PPI networks, based on Thompson Tau test. In addition SQSTM1/p62 had the highest harmonic centrality in ALS+FTD group, and UBC presented in both groups as the only none ALS-causative protein. Interestingly the widely presented C9ORF72 mutation (34% fALS and 7% sALS[48, 49]) was not top ranked in either group. However, its interactive protein profile was unique from other ALS-causative proteins (Fig 4A).

### 3.5 Functional enrichment analysis

GO term and KEGG pathway enrichment analysis were performed on the 393 clusters, k = 3 in classical ALS group as well as the 578 clusters, k = 3 in ALS+FTD group. GO term analysis was carried out in three categories, including biological processes (BP), molecular function (MF), and cellular components (CC). Tables 4 and 5 describe the top five GO terms of BP, MF, and CC as well as top five pathways in KEGG (GO terms with p values <0.01 were discarded). In classical ALS group the most significant terms in BP and CC were all RNA processing related; the translation control was also highlighted in BP (Table 4). In accordance with the results from GO terms analysis, ribosme and spliceosome pathways were all significant in KEGG pathway analysis (Table 5). Consistenly, the ALS+FTD group showed high functional enrichment in translation control, ribosome and RNA processing (Table 4). Autophagy, ribosome, spliceosome and neurotrophin signaling pathways were shared by classical ALS and ALS+FTD groups in KEGG enrichment analysis (Table 5).

**Table 4:** Most significantly enriched GO terms.

	Classical ALS		ALS+FTD	
	Terms	P *value*	Terms	P *value*
Biological	mRNA metabolic process	7.12E-23	Translational elongation	9.54E-45
Process	mRNA processing	5.40E-21	Translation	8.27E-27
	RNA processing	5.50E-21	mRNA metabolic process	5.95E-25
	Translational elongation	1.00E-19	RNA processing	5.80E-24
	RNA splicing	1.17E-17	mRNA processing	2.17E-23
Cellular	Ribonucleoprotein complex	6.71E-45	Ribonucleoprotein complex	4.76E-62
Component	Organelle lumen	4.56E-28	Cytosol	1.74E-54
	Intracellular organelle lumen	1.15E-27	Cytosolic part	3.09E-38
	Membrane-enclosed lumen	2.60E-27	Intracellular organelle lumen	3.82E-34
	Cytosol	7.40E-27	Organelle lumen	4.39E-34
Molecular	RNA binding	1.06E-38	RNA binding	3.40E-48
Function	Nucleotide binding	9.80E-16	Structural constituent of ribosome	1.34E-29
	Enzyme binding	3.33E-15	Nucleotide binding	6.21E-22
	Structural constituent of ribosome	2.57E-12	Structural molecule activity	2.23E-20
	Unfolded protein binding	6.83E-11	Enzyme binding	4.20E-18

**Table 5:** Most significantly enriched KEGG pathways.

Classical ALS		ALS+FTD	
Terms	*P* value	Terms	*P* value
1. Ribosome 2. Regulation of autophagy 3. Spliceosome 4. mTOR signaling pathway 5. Neurotrophin signaling pathway	3.94E-14 1.33E-07 1.62E-07 6.97E-06 6.39E-05	1. Ribosome 2. Neurotrophin signaling pathway 3. Prostate cancer 4. Regulation of autophagy 5. Spliceosome	2.81E-29 4.73E-10 9.69E-09 1.91E-08 1.26E-06

### 3.6 Common proteins analysis

To investigate how these functionally diverse pathogenic proteins all led to motoneuron degeneration, we analyzed their PPI profiles with the aim to identify common ground downstream of ALS-causative proteins. For the classical ALS group, in addition to the 78 interacting proteins at k = 4 (Fig 2A), we included proteins involved in significant GO terms and KEGG pathways as protein set *S1*. For the ALS+FTD group, 27 proteins from k = 5 (Fig 2B) along with proteins from significant GO terms and KEGG pathways formed protein set *S2*. The DFloyd algorithm was employed to investigate the interaction between ALS-causative protein set (*T)* and downstream protein sets (*S1 and S2*). The results suggested that UBC was the most interconnected protein in both classical ALS and ALS+FTD groups. Other top ranking common proteins shared by both groups were YWHAZ, PARK2, GBAS and GABARAP (Table 6). The detailed interactive profiles of selective proteins were shown in Fig 4B, in which UBC was connected to all ALS-causative proteins except ANG and CHCHD10.

**Table 6:** Downstream proteins ranked by number of direct interactions with ALS-causative proteins.

Classical ALS				ALS+FTD			
Rank	Uniprot	Protein	Interacting #	Rank	Uniprot	Protein	Interacting #
1	POCG48	UBC	19**	1	POCG48	UBC	22**
2	Q13501	SQSTM1	11*	2	P63104	YWHAZ	10
3	P63104	YWHAZ	9	3	O60260	PARK2	9
4	Q00987	MDM2	8	4	O75323	GBAS	9
5	O00443	PIK3C2A	8	5	O95166	GABARAP	9
6	O60260	PARK2	8	6	P11021	GRP78	9
7	O75323	GBAS	8	7	P55854	SUMO3	9
8	O95166	GABARAP	8	8	Q13618	CUL3	9
9	P55854	SUMO3	8	9	Q9H492	MAP1LC3A	9
10	Q13618	CUL3	8	10	Q00987	MDM2	9
11	P60520	GABARAPL2	8	11	Q9Y4P8	WIPI2	8
12	Q86VP9	PRKAG2	7	12	Q676U5	ATG16L1	8
13	Q9BSB4	ATG101	7	13	Q14999	CUL7	8
14	Q9H1Y0	ATG5	7	14	Q15843	NEDD8	8
15	Q9H492	MAP1LC3A	7	15	P60520	GABARAPL2	8
16	P11021	GRP78	7	16	Q9H0R8	GABARAPL1	8
17	Q676U5	ATG16L1	7	17	O00443	PIK3C2A	8
18	Q9H0R8	GABARAPL1	7	18	Q9H1Y0	ATG5	8
19	P61956	SUMO2	7	19	P61956	SUMO2	8
20	Q14999	CUL7	7	20	Q13616	CUL1	8
21	Q15843	NEDD8	7	21	Q9BSB4	ATG101	7
22	Q9Y4P8	WIPI2	7	22	Q9UGJ0	PRKAG1	7
23	Q13573	SNW1	6	23	P07437	TUBB	7
24	Q99459	CDC5L	6	24	P07900	HSP90AA1	7
25	P15297.	NOF	6	25	Q13573	SNW1	7
26	P54619	PRKAG1	6	26	Q86VP9	PRKAG2	7
27	Q13286	CLN3	6	27	P54619	PRKAG1	7
28	Q9GZZ9	UBA5	6	28	Q13286	CLN3	7
29	Q9NR46	SH3GLB2	6	29	Q9GZZ9	UBA5	7
30	Q9Y484	WDR45	6	30	Q9NR46	SH3GLB2	7
31	Q9UGJ0	PRKAG2	6	31	Q99459	CDC5L	6
32	O75147	OBSL1	5	32	P15297	NOF	6
33	O75530	EED	5	33	Q9Y484	WDR45	6
34	P02751	FN1	5	34	O75147	OBSL1	6
35	P13612	ITGA4	5	35	O75530	EED	6
36	P19320	VCAM1	5	36	P02751	FN1	6
37	P27694	RPA1	5	37	P13612	ITGA4	6
38	P35244	RPA3	5	38	P19320	VCAM1	6
39	P35638	DDIT3	5	39	P35638	DDIT3	6
40	P54646	PRKAA2	5	40	P54646	PRKAA2	6
41	Q15831	STK11	5	41	Q15831	STK11	6
42	Q13616	CUL1	5	42	P62993	GRB2	6
43	O15530	PDPK1	5	43	P27694	RPA1	6
44	P62993	GRB2	5	44	P27361	MAPK3	6

** >mean ± τ ∙ SD.

* >mean ± (τ ∙ SD)/2.

## Discussion

### Essential proteins

The ECC method was used to identify essential proteins in PPI network that integrated global topological properties and cluster information[43]. Our results showed that VCP, SQSTM1/p62, hnRNPA1, FUS and TDP-43 were proteins with significantly high harmonic centrality in ECC method. VCP is known to associate with some forms of FTD, Paget's disease of the bone (PDB) and inclusion body myopathy (IBM) before the discovery that mutations in these same genes account for approximately 1–2% fALS patients[50, 51]. The finding of *VCP* mutations in ALS gives rise to an interesting phenomenon that the mutations on single gene can affect multiple tissues and result in distinctive diseases. VCP is associated with nucleocytoplasmic transport and putative ATP binding protein[52]. Bartolome *et al*. showed that *VCP* mutations were likely resulted in reduced ATP level in energy production due to dysregulated mitochondria[53], which might partially explain the profound effects of mutations in diverse tissues. The idea of “multisystem proteinopathy” is further reinforced by the discovery of *HNRNPA1/HNRNPA2B1* mutations in some forms of ALS patients[54]. Firstly the involvement of hnRNPA1 brings up the importance of RNA processing in motoneuron degeneration that was previously highlighted by discovery of mutations of FUS and TDP-43 being implicated in both ALS and FTD[55–58]. Secondly the results suggest that certain ALS subtypes indeed have wide-spreading disease effects on not only motoneuron but also on muscles, brain and bones. These wide-spread effects imply that motoneuron degeneration in ALS, at least in some subtypes, might be the final outcome of a series of genetic deficits causing multisystem dysregulations instead of a single disease. Given above rationale that ALS might not be a single disease as well as the complexity of motoneuron degeneration etiology, it is reasonable to integrate diverse causative proteins to identify a hub network in which essential proteins strongly interact with each other and downstream proteins (Fig 2A and 2B). Such a hub network would ideally incorporate as many causative proteins as possible. Thus, essential proteins in the network provide important clues to understand mechanisms underlying motoneuron degeneration. SQSTM1/p62 was identified as having involvement in ALS, FTD and PDB[18, 59, 60]. P62, *SQSTM1* encoding protein, has been reported to be strongly associated with ubiquitination and involve in autophagy, oxidative stress and NF-ƙB pathway[59, 61, 62]. In ALS and FTD, it is frequently found in inclusion bodies containing polyubiquitinated proteins along with UBQLN2[63]. The common pathological features of ALS and FTD strongly imply the overlapping between diseases spectrum[64]. *TARDBP* encoded protein, TDP-43, is found in the common pathological hallmark, ubiquitin-positive inclusion bodies, in both ALS and FTD[56]. TDP-43 is an important regulator of RNA metabolism and its association with ALS evokes major interest in the role of RNA processing in ALS[65]. Soon after the discovery, *FUS* mutations were also found in small cohort of fALS patients (~4%)[55, 58]. More importantly the majority of mutants are clustered at RNA-binding domain rich C-terminus, a feature similar to TDP-43 in ALS. However, the *FUS* mutation induced ALS seems to lack of TDP-43 positive inclusions. Further evidence showed that overexpressed wild-type FUS rescued *TARDBP* knockdown, but not vice versa, suggesting TDP-43 might be upstream of FUS[66]. In current work, we calculated the essential proteins based on PPI network built from ALS causative proteins. hnRNPA1, TDP-43 and FUS are all associated with RNA-processing while VCP and SQSTM1/p62 are involved in nucleocytoplasmic transport and autophagy respectively. It seems that we have a chain of essential proteins involved in cellular activities ranging from nucleus/RNA level to cytoplasmic/protein level. It is clear that this chain tightly regulates protein turnover activities through upstream RNA metabolism to downstream protein chaperoning and clearance. Conceivably, disruption in any part of the chain would lead to catastrophic results. However, certain mutations only affect very small amount of ALS patients, which does not to fit in the so called “centrality-lethality” theory in PPI network analysis field. The contradiction is likely due to redundancies of regulatory proteins, compensation pathways or feedback regulations. Besides it is unclear why the same mutation would cause different levels of damage in tissues, and resulted in distinctive phenotypes, onset time and disease severity. One possible explanation is that each tissue has its own protein homeostasis/turnover profile, such that each mutation is likely to cause different level of impacts onto the PPI network. Thus, identifying essential proteins and hub network, and a detailed profiling of interested tissue are worthy further investigation to precisely assess the level of damage in specific tissue due to certain mutations. In addition, mutations of essential proteins with various functions all led to motoneuron degeneration in ALS suggests that there might be common downstream proteins where different pathogenic mechanisms converge.

### Downstream common proteins

In present work, we analyzed both classical ALS and ALS+FTD PPI networks, and identified the aforementioned essential proteins. Further calculation revealed a set of common downstream proteins with strong interactivities toward causative proteins. In the classical ALS group, UBC and SQSTM1/p62 stood out as the most interconnected downstream proteins. *SQSTM1* as a causative gene is categorized in the ALS+FTD group but not the classical ALS group; the result validates our algorithm in identifying potential targets associated to motoneuron degeneration. There are a set of widely-interconnected downstream proteins shared by both groups: UBC, YWHAZ, PARK2, GBAS and GABARAP (Table 6). It seems reasonable to consider UBC as the most interconnected downstream protein in both classical ALS and ALS+FTD groups since it is a polyubiquitin precursor protein. However, it is intriguing that ubiquitin A-52 (UBA-52) and polyubiquitin B (UBB) are not on top of the list; especially as UBB is involved in several neurodegenerative diseases. *UBB* frame shifting mutation (*UBB*+1) impedes proteasomal proteolysis, and has been extensively identified in Alzheimer disease (AD), FTD and Huntington disease (HD) as pathological hallmark[67]. However, *UBB*+1 transgenic mice with mutant huntingtin showed no aberrant phenotype except increased inclusion bodies, albeit they were more sensitive to the toxicity[68]. In contrast to the well-studied role of UBB in neurodegeneration, to the best of our knowledge, there is no strong molecular evidence to link UBC and neurodegeneration to date. However, the same observation, high interactivities between UBC and causative proteins in neuronal degeneration, was also made in an *in silico* network analysis of AD[69]. *UBC*, not to be confused with ubiquitin-conjugating enzyme (also called UBC or more frequently E2), along with *UBB* encode polyubiquitin precursor proteins with nine and three ubiquitin tandem repeats respectively. Both *UBB* and *UBC* transcription is shown to be induced and upregulated in response to various cellular stresses[70–74], in addition to the constitutive expression under normal condition[75]. The presumed redundancy of both genes seems to be insufficient to compensate for the loss of each other. In the case of *Ubc* knockout mice, it is lethal to transgenic mice at embryonic stage due to the disrupted fetal liver development[75, 76]. On the other hand, Ubb-null mice lead to damaged neurons within the arcuate nucleus of the hypothalamus[77]. The results strongly suggest that UBC and UBB are not functionally redundant. Since UBB and UBC are functionally and structurally similar, the role and importance of UBB regulation in AD might reveal the importance of UBC in neurodegeneration. UBB+1 and UCHL1 are both important regulators, likely to have opposite effects, of beta-amyloid production and amyloid precursor protein processing in AD [78]. Although UCHL1 is not known to directly cause ALS, UCHL1 null mice showed upper motoneuron vulnerability [79]. Thus it stressed the importance of protein quality control which involved both UCHL1 and polyubiquitin precursor. Moreover UBC demonstrates a tissue-specific manner in coping with various cellular stresses instead of a generalized response[72, 80]. UBC is shown to be positively regulated by Sp1, MEK1 and FOXO3a in rat L6 muscle cell[80–82], and FOXO3a is neuroprotective in models of motoneuron diseases[83]. Additionally TDP-43 regulates protein quality control through FOXO-dependent pathway[84]. Thus it is highly plausible that FOXO-mediated UBC expression contributes to motoneuron proteasome regulation which eventually affects ubiquitin-proteasome system. Given that UBC is able to interact with almost all ALS causative proteins (22 out of 24), further investigation is needed to profile UBC in motoneuron to better understand the behavior of this common downstream protein in ALS. Another interesting finding is the presence of 14-3-3 protein family member (YWHAZ) in downstream protein analysis. It has been known that 14-3-3 protein interacts with TDP-43 and SOD1 in ALS to modulate neurofilament light chain mRNA stability in G93A and A4T mSOD1 mice[85]; 14-3-3 protein was also found in lewy body in sALS[86]. YWHAZ also interacts with FUS in ALS whereas its isoform YWHAQ was reported to have significantly elevated mRNA level in sALS patients[86, 87]. 14-3-3 protein extensively involves in apoptosis, and protein and mRNA stabilization, however, much remains unknown about its role in ALS pathology. Notably its isoform, YWHAE, also presents in the results of cluster analysis and ECC, which suggests a deep involvement of this protein family in ALS.

### C9ORF72

Although considerable progression has been made in identifying ALS causative genes over past few years, *TARDBP*, *FUS*, *VCP*, *UBQLN2*, *SQSTM1* and *C9ORF72* for example, underlying mechanisms remain elusive, as does the clinical spectrum of phenotypes. C9ORF72 not only associates with a large portion of ALS patients (7% sALS/ 34% fALS) but also implicates in FTD (25%). The functions of *C9ORF72* encoded protein are not fully clear, however, it has been shown capable of interacting with hnRNPA1, hnRNPA2/B1, ubiquilin-2 in immunoprecipitation from cell line models[88]. The GGGGCC repeats in *C9ORF72* translate to dipeptide repeat protein (DPR) in repeat-associated non-ATG manner which could impair RNA processing and lead to cell death[89, 90]. Recent study showed that C9orf72 mutation led to SQSTM-1 (p62) pathology as seen in ALS/FTD patients through Rab1a and ULK1 autophagy initiation complex [91]. Our analysis of C9ORF72 interacting profile is based on I2D database which does not reflect the DPR proteins and PPI described above. This should be taken as caveat in interpreting our analysis results. Nevertheless, the functional enrichment analysis suggests that ALS+FTD is different from classical ALS in GO terms and KEGG pathway. Specifically, unfolded protein response (UPR), membrane transport, splicing and RNA metabolism pathway are unique in ALS+FTD group compared to classical ALS, which highly enriches with autophagy, survival and nutrient-sensing pathways (Table 6). The result is consistent with brain transcriptome study in ALS conducted by Prudencio *et al*., in which C9ALS mainly affected intracellular transport/localization and UPR pathways, and sALS involved cytoskeleton organization, defense response and synaptic transmission while alternative splicing and RNA processing defects were found in both[30]. In addition, the surprisingly low ranking in centrality and topology analysis of C9ORF72 suggests that certain forms of ALS associated with FTD might not experience exactly the same molecular mechanism as classical ALS, although they share many pathological hallmarks and final destiny. If this holds true, future ALS therapeutic development might take into consideration individual genetic profile to tailor treatment.

## Conclusion

The PPI network analysis highlights a set of ALS causative proteins as essential proteins, which form a complete regulatory chain of protein turnover. The result emphasizes on the importance of protein turnover in motoneuron degeneration. More importantly the hub network formed by essential proteins provides a converging point connecting other ALS causative proteins to downstream common proteins. It might help to explain why these functionally diverse ALS mutations all led to motoneuron degeneration. Our *in silico* analysis suggests a more active role of UBC in motoneuron degeneration which has been overlooked. Considering its active regulatory roles in ubiquitination and transcription under various conditions, UBC is likely the common protein connecting most causative proteins to proteasome regulation. UBC itself might not be sufficient to cause motoneuron degeneration, but it surely can serve as a useful start point to explore further UBC-related pathways that might shed light on common mechanism underlying motoneuron degeneration.

## Supporting information

S1 Text (TXT)

S2 Text (TXT)
